# Altered Sleep Patterns in Wilson’s Disease Including Shortened REM Latency

**DOI:** 10.3390/diagnostics15212771

**Published:** 2025-10-31

**Authors:** Jan Paweł Bembenek, Tomasz Litwin, Agnieszka Antos, Wojciech Jernajczyk

**Affiliations:** 1 Department of Clinical Neurophysiology, Institute of Psychiatry and Neurology, Sobieskiego 9, 02-957 Warsaw, Poland; wjernajczyk@ipin.edu.pl; 2 Department of Neurology, Stroke Unit and Rehabilitation Subunit, Wolski Hospital, 01-211 Warsaw, Poland; tomlit@medprakt.pl; 3 Second Department of Neurology, Institute of Psychiatry and Neurology, 02-957 Warsaw, Poland; agnieszkaantos91@wp.pl

**Keywords:** Wilson’s disease, sleep disturbances, narcolepsy, polysomnography, copper

## Abstract

**Background and Clinical Significance:** Wilson’s disease (WD) is an inherited, multisystem disorder of copper metabolism, resulting in pathological copper accumulation in various tissues (predominantly the liver and brain) and leading to secondary organ damage and corresponding clinical manifestations. Sleep disorders are frequent in neurodegenerative disorders, but remain underdiagnosed and poorly characterized in WD. **Case presentation:** We describe the case of a 51-year-old patient with WD presenting predominantly with neurological symptoms, who underwent routine video-polysomnography (v-PSG). The examination revealed shortened sleep latency, reduced rapid eye movement (REM) sleep latency, and sleep fragmentation—features of sleep architecture frequently observed in narcolepsy. These abnormalities worsened at follow-up despite the introduction of anti-copper treatment and concomitant neurological improvement. However, the patient did not report clinical symptoms of narcolepsy, and none were confirmed by the evaluating sleep specialist. **Conclusions:** This case highlights that sleep disorders (SDs) are common in patients with WD. Such patients may experience a wide range of SDs, and anti-copper treatment may improve sleep quality in addition to alleviating neurological symptoms. Narcolepsy is a rare but possible manifestation of SDs in WD. Therefore, whenever symptoms suggestive of sleep disturbances occur, WD patients should be referred to a sleep specialist, as accurate diagnosis and targeted treatment may profoundly improve quality of life, daily functioning, and long-term disease management.

## 1. Introduction

Wilson’s disease (WD) is an inherited, multisystem disorder of copper metabolism, characterized by pathological copper accumulation in various tissues (predominantly the liver and brain, but potentially affecting all organs), leading to secondary organ damage and corresponding clinical manifestations (mainly hepatic and/or neuropsychiatric) [1,2,3]. WD presents with a wide spectrum of symptoms. The phenotypic presentation may involve isolated liver disease (rarely) or a mixed hepatic and extrahepatic form, most often with neuropsychiatric features [1,2]. Neurological manifestations of WD include movement disorders such as tremor, dystonia, chorea, and ataxia, often accompanied by gait and posture disturbances, drooling, and dysarthria [4,5,6]. Psychiatric symptoms are not pathognomonic for WD and may include behavioral and personality changes, mood disorders, cognitive impairment, as well as anxiety, psychosis, anorexia, and, less commonly, other psychiatric syndromes [4,6].

With respect to the neuropsychiatric spectrum, increasing attention has recently been directed toward sleep disturbances (SDs), which have been reported in up to 80% of WD patients [7,8,9,10,11,12,13,14,15]. The high prevalence of SDs in WD may be explained by coexisting liver disease (involving disturbances of melatonin, ammonia, and glucose metabolism, thermoregulation, and ghrelin secretion) [8], as well as brain copper accumulation in multiple structures (particularly the basal ganglia), resulting in secondary astrogliosis, neuronal necrosis, neurodegeneration, and possibly pineal gland dysfunction (atrophy) [9,10,11,12]. Copper may accumulate in various brain structures, some of which are crucial for sleep regulation. The hypothalamic orexin neurons, basal forebrain, and brainstem arousal nuclei play key roles in the control of REM sleep as components of a complex arousal network. The hypothalamic orexin system promotes wakefulness and suppresses REM sleep by modulating arousal centers in the brainstem and basal forebrain. The basal forebrain is essential for attention, while the brainstem nuclei contain the core circuitry underlying REM sleep. Copper accumulation in these regions may disrupt neural circuits that regulate the sleep–wake balance and contribute to sleep disturbances [16]. However, the mechanisms underlying copper accumulation in brain regions involved in sleep regulation remain poorly understood.

Clinically, SDs in WD have been reported as insomnia, excessive daytime sleepiness and hypersomnia, restless legs syndrome, cataplexy, and rapid eye movement (REM) sleep behavior disorder [7]. If untreated, SDs may have serious consequences, including depression, progressive neurodegeneration, and reduced quality of life (QoL) [6,7].

To date, only a few studies on SDs in WD have used video-polysomnography (v-PSG) to objectively assess their frequency; most available data come from questionnaire-based studies, which are prone to high bias [10,11,12,13,14,15]. Notably, no cases of narcolepsy or short-onset REM periods (SOREMPs) have been described in WD so far [17,18].

The aim of our report is therefore to present a WD patient requiring differential diagnosis between narcolepsy and SOREM in order to highlight the importance of v-PSG in the evaluation of SDs in WD.

## 2. Case Report

**Figure 1:**
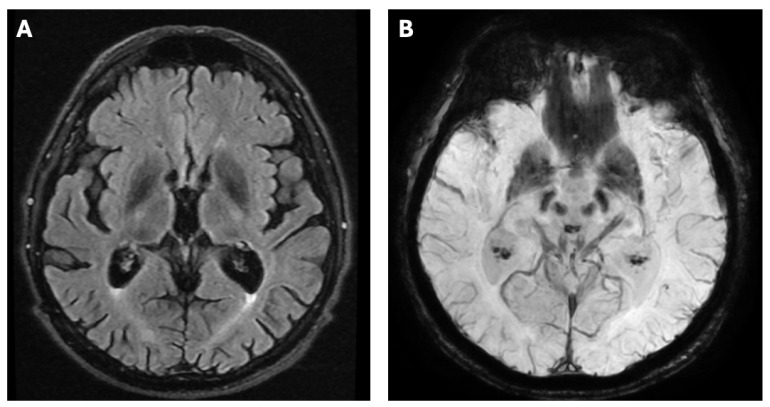
Brain MRI of the patient showing reduced signal intensity symmetrically in the putamen, globus pallidus, and caudate nuclei on the T2-weighted sequence (**A**); reduced signal intensity symmetrically involving the putamen, globus pallidus, red nuclei, and substantia nigra on the SWI sequence (**B**).

**Figure 2:**
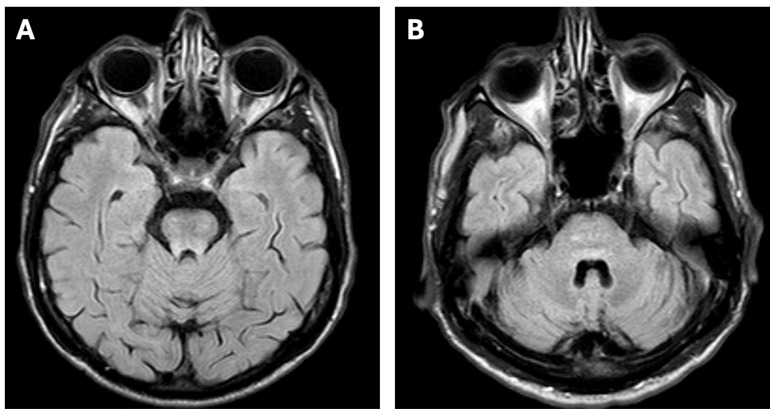
Brain MRI of the patient showing increased signal intensity in the pons (**A**) and midbrain (**B**) on the T2-weighted sequence.

**Figure 3:**
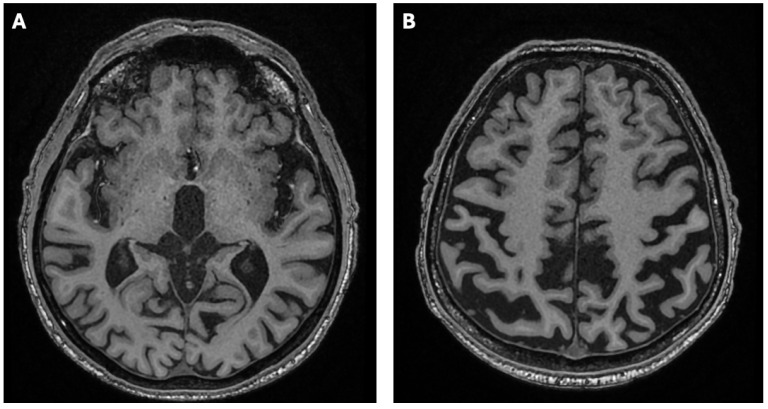
Brain MRI of the patient showing dilatation of the third ventricle on the T1-weighted sequence (**A**) and cortical atrophy (**B**).

A 51-year-old man was diagnosed with WD in 2016 during the differential diagnostic workup of parkinsonian symptoms. Since 2014, he had gradually developed neurological manifestations, including gait disturbances, upper limb tremor, dysarthria, and hypomimia. These features initially led to a diagnosis of Parkinson’s disease in 2015. Treatment with ropinirole and levodopa was initiated; however, the patient failed to show meaningful clinical improvement, which prompted further evaluation and referral to the neurology department. During hospitalization at the Second Department of Neurology, Institute of Psychiatry and Neurology in Warsaw, in April 2016, neurological examination confirmed hypomimia, dysarthria, postural tremor of the upper limbs, impaired rapid alternating movements, gait disturbance, and abnormal postural reflexes. He scored 40 points on the Unified Wilson’s Disease Rating Scale (UWDRS)—6 points in part II (activities of daily living) and 36 points in part III (detailed neurological examination). No cognitive deficits were detected on neuropsychological evaluation. Laboratory tests demonstrated thrombocytopenia (125 × 10^3^/µL [normal: 150–400]). Brain magnetic resonance imaging (MRI) performed at that time revealed reduced T2-weighted and Susceptibility-Weighted Imaging (SWI) signal intensity symmetrically involving the putamen, globus pallidus, caudate nuclei, red nuclei, and substantia nigra (Figure 1A,B), with concomitant increased signal intensity in the midbrain and pons on T2-weighted images (Figure 2A,B), as well as cortical atrophy with dilatation of the third ventricle on T1-weighted sequences (Figure 3A,B).

**Figure 4:**
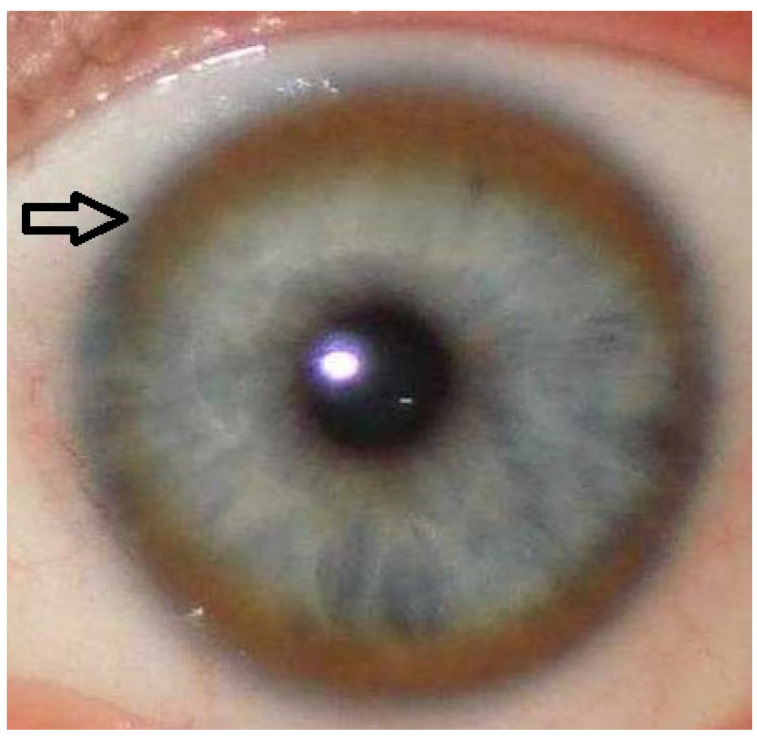
Kayser–Fleischer (K–F) ring visible as a brown corneal ring (black arrow).

Abdominal ultrasound demonstrated a heterogeneous liver echotexture with the presence of regenerative nodules, while the spleen appeared unremarkable. The diagnosis of WD was confirmed on the basis of abnormal copper metabolism parameters, including decreased serum ceruloplasmin (13.3 mg/dL [normal: 20–60]), elevated non-ceruloplasmin bound copper (NCC) calculated indirectly (16.1 μg/dL [normal: 5–15]), and increased 24-h urinary copper excretion (475 μg/24 h [normal: <50]). Additional supportive findings included the presence of a Kayser–Fleischer ring detected on slit-lamp examination (Figure 4), and genetic testing confirming homozygous *ATP7B* mutations. Treatment with zinc salts (zinc sulfate, 180 mg elemental Zn^2+^ daily) was initiated.

Over the following years, substantial neurological improvement was observed, with only mild residual symptoms persisting, such as discrete gait disturbances and hypomimia. His UWDRS score decreased to 6 points (0 in part II and 6 in part III). Monitoring of copper metabolism indicated effective anti-copper therapy with zinc salts, as shown by NCC levels of 9.1 μg/dL (therapeutic range: 5–15) and a 24-h urinary copper excretion of 65 μg/24 h (therapeutic range: <75 μg/24 h according to recommendations [1]) without any treatment-related adverse events. In 2016, the patient underwent video-polysomnography (v-PSG), followed by a routine re-examination 20 months later. Before the examination, the patient underwent standard preparation: maintaining a regular sleep schedule (bedtime between 10:30–11:30 p.m. and waking at 7 a.m.), no history of night-shift work, and no daytime naps on the day preceding the study. The last cup of coffee was consumed in the morning of the day before the examination. The patient did not use stimulants, hypnotics, or psychoactive drugs. There was no history of insomnia, circadian rhythm disorder, or obstructive sleep apnea. The patient also did not report excessive daytime sleepiness (EDS), as assessed through a clinical interview. Depression was excluded based on psychiatric evaluation supplemented with the Beck Depression Inventory. In both studies, most sleep parameters were abnormal, and further deterioration was observed at follow-up. Sleep efficiency (SE) declined from 73.5% to 67.4%, with a reduction in both stage N2 sleep (167.5 min, 47.3% vs. 112 min, 34.2%) and stage N3 sleep (36 min, 10.2% vs. 24 min, 7.3%). Sleep latency, initially short at 4.5 min, normalized at follow-up (11 min). The most notable abnormalities concerned rapid eye movement (REM) sleep latency and wake after sleep onset (WASO). REM latency was markedly reduced in both studies (5 min vs. 19.5 min), whereas WASO values were significantly elevated (123.5 min vs. 147 min). Interestingly, REM sleep duration increased at follow-up (66.0 min, 18.6% vs. 106.3 min, 32.5%). Detailed results of both v-PSG examinations are summarized in Table 1.

**Figure 5:**
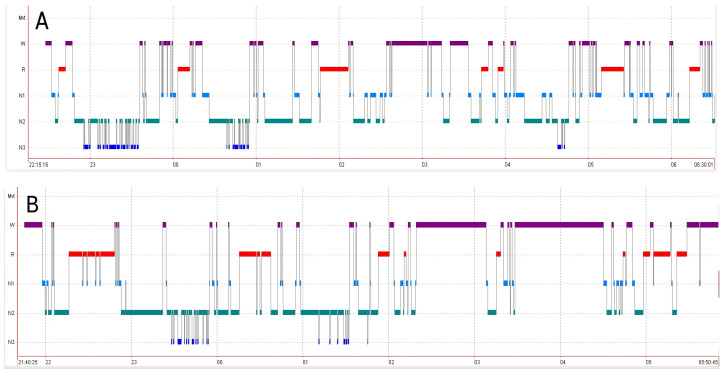
Hypnogram before treatment initiation (**A**) and at follow-up (**B**). W, wake; R, REM sleep; N1, non-REM stage 1; N2, non-REM stage 2; N3, non-REM stage 3.

Interestingly, this patient exhibited shortened sleep latency, reduced REM sleep latency, and sleep fragmentation in both v-PSG assessments—features of the sleep architecture frequently observed in narcolepsy. These parameters further deteriorated at follow-up. However, the patient did not report any clinical symptoms of narcolepsy, nor were such symptoms confirmed by the sleep specialist who evaluated him. Moreover, the patient had no history of sleep disturbances prior to the onset of WD symptoms. Unfortunately, cerebrospinal fluid orexin (hypocretin) levels were not assessed. Prior to both vPSG examinations, the Beck Depression Inventory was administered to exclude depression. The patient will continue to be monitored in our department. Hypnograms before and after treatment introduction are presented in Figure 5.

## 3. Discussion

Although our patient exhibited some polysomnographic features suggestive of narcolepsy, he did not fulfill its clinical and electrophysiological diagnostic criteria [19]. Therefore, further diagnostic procedures, such as the Multiple Sleep Latency Test (MSLT) and cerebrospinal fluid orexin level assessment, were not performed. This limitation reduced the diagnostic sensitivity in our case. Nonetheless, MSLT would have been particularly valuable in confirming or excluding narcolepsy. Narcolepsy is the most well-recognized disorder associated with excessive daytime sleepiness (EDS) as the predominant symptom, and it significantly increases the risk of motor vehicle accidents, accidental injuries, and occupational difficulties [19,20]. The classic narcoleptic pentad comprises EDS, cataplexy, sleep paralysis, hypnagogic or hypnopompic hallucinations, and sleep fragmentation [21]. In addition, narcolepsy is frequently accompanied by a wide range of cognitive, metabolic, motor, autonomic, and psychiatric disturbances [22,23]. Importantly, our patient did not report any of the typical clinical symptoms of narcolepsy.

V-PSG remains the gold standard for objectively assessing both the quantity and quality of sleep [24]. In our patient, polysomnography revealed an increase in REM sleep duration at follow-up after treatment initiation. In the first study, a short REM sleep latency of only 5 min was recorded. Although a REM latency below 15 min is considered highly specific for narcolepsy according to current diagnostic criteria [25], such isolated findings are rare in general sleep clinic populations (<1%) [26]. The diagnosis of narcolepsy, however, also requires the presence of characteristic clinical symptoms, which were absent in this case. Unfortunately, no MSLT was performed, nor were hypocretin levels evaluated. It should also be emphasized that a shortened REM sleep latency may occur more frequently in patients with depression [25] as well as in neurodegenerative disorders [27]. To date, no large-scale studies have systematically assessed the prevalence of such sleep abnormalities in WD patients.

Short-onset REM periods (SOREMPs), defined as REM sleep occurring within minutes of sleep onset, are not observed in healthy individuals and have traditionally been considered highly suggestive of narcolepsy [24,28,29]. Patients with SOREMPs often experience poor sleep continuity with frequent nocturnal awakenings. However, more recent studies indicate that SOREMPs are not pathognomonic and may occasionally appear in other clinical conditions, including circadian rhythm disorders, severe sleep deprivation, obstructive sleep apnea, and various neurodegenerative diseases [24,26,30]. Their presence is therefore best interpreted in the context of the overall clinical picture and complementary diagnostic testing, such as MSLT and hypocretin level assessments. In WD, the occurrence of SOREMPs may reflect underlying disruption of brainstem and hypothalamic structures involved in REM regulation, although robust data in this population remain lacking. In our patient, a potentially beneficial finding was the prolongation of REM sleep duration after initiation of anti-copper treatment (66.0 min, 18.6% vs. 106.3 min, 32.5%). During REM sleep, the activity of histaminergic, noradrenergic, and serotonergic neurons is markedly suppressed. It has been postulated that reduced activity during REM facilitates the synthesis of these monoamines and promotes repair of their receptors [28,29]. Based on this, we hypothesize that the introduction of anti-copper therapy may have supported regenerative processes within histaminergic, noradrenergic, and serotonergic systems, despite the lack of improvement in other v-PSG parameters.

In patients with WD and sleep disturbances, anti-copper therapy may not only improve neurological symptoms but also positively affect sleep parameters and overall sleep quality [30]. Nevertheless, in our patient, no improvement was observed in most v-PSG parameters at follow-up after treatment initiation. This suggests that not all types of sleep disturbances in WD are reversible, and that shortened REM sleep latency may represent a particularly resistant abnormality. The observed prolongation of REM sleep duration, however, may indicate ongoing restorative adaptations within sleep architecture in this patient. We also suspect that patient’s sleep abnormalities could represent coexisting primary sleep disorders rather than secondary manifestations of WD.

The v-PSG in our patients revealed shortened sleep latency, reduced REM sleep latency, and sleep fragmentation—findings contrary to other v-PSG studies in WD, which predominantly reported prolonged sleep-onset and REM latencies with reduced proportions of REM sleep [7,11,30]. These discrepancies may result from the small number of WD patients analyzed with v-PSG to date, as well as from the broad spectrum of clinical manifestations associated with WD. Copper accumulation varies across tissues—with typically higher concentrations in the basal ganglia and liver—and may also differ among brain regions responsible for sleep regulation. Additional factors such as medication effects, mood state, or circadian influences might further contribute to these divergent REM findings. As our patient had no psychiatric comorbidities and did not use any drugs affecting neurotransmission, his hypnogram findings are particularly intriguing.

At present, single-case reports and studies evaluating sleep disturbances in WD remain severely limited. Future research should emphasize v-PSG assessment as an objective tool in all WD patients reporting sleep problems, rather than relying predominantly on questionnaires as in most studies conducted to date. Notably, improvement in sleep quality following anti-copper treatment has only been evaluated in one study by Jernajczyk et al. [30].

Another promising area that deserves further investigation is the potential use of melatonin in WD [30]. Several studies have discussed its possible role, both in copper metabolism and in the management of WD-related sleep disturbances [31,32,33,34,35,36]. This hypothesis is based on the ability of melatonin metabolites—particularly cyclic 3-hydroxymelatonin (3OHM) and *N*-acetyl-*N*-formyl-5-methoxykynuramine (AFMK)—to act as copper chelators while simultaneously exerting antioxidant effects. These mechanisms may be especially relevant given the role of oxidative stress in the pathogenesis of psychiatric and neurocognitive symptoms in WD [17]. Moreover, melatonin is increasingly prescribed as a safe and well-tolerated treatment for various sleep disorders [30,31], suggesting that it could provide a dual benefit in patients with WD by improving sleep quality and potentially modulating disease-related oxidative stress. However, our patient did not receive melatonin, and therefore, we cannot exclude the possibility that its administration might have improved sleep parameters. Further studies are needed to clarify its efficacy and establish evidence-based recommendations for melatonin use in WD.

## 4. Study Limitations

We present a single case report of a patient with WD and SDs. This study has several limitations. First, we did not extend the diagnostic evaluation to include the MSLT or cerebrospinal fluid orexin level assessment. Second, the patient underwent only a single night of v-PSG recording. This may not provide sufficient reliability for assessing sleep parameters, as night-to-night variability can lead to chance findings or “first-night effects.” Previous studies have demonstrated that multiple-night recordings improve the stability of sleep parameters and reduce measurement errors [36,37,38]. Therefore, this single-night examination may not fully capture the variability in sleep architecture and limits the ability to infer a causal relationship between anti-copper therapy and the observed changes in REM parameters. Finally, as this is a single-case report, the findings are hypothesis-generating and cannot be generalized to the broader population of WD patients. We also did not apply standardized tools to assess excessive daytime sleepiness.

## 5. Conclusions

Diagnosing and treating sleep disorders is challenging, and their impact on patients is often difficult to quantify. Such disturbances can significantly impair quality of life. The present case report aims to highlight that sleep disorders may also occur in patients with WD. These patients may experience a range of sleep disturbances, and anti-copper treatment may not only alleviate neurological symptoms but also improve sleep quality [30,31]. In the presence of symptoms suggestive of sleep problems, referral to a sleep specialist is recommended as accurate diagnosis and appropriate management can profoundly improve quality of life and functional outcomes [32,33,34,35,36].

## Figures and Tables

**Table 1:** Polysomnography parameters in first and follow-up examination.

	Polysomnography	
Parameter	Baseline	Follow-Up Examination
Total sleep time, min.	354.3 (100%)	327.3 (100)
Stage 1N, min	84.8 (23.9%)	85 (26)
Stage 2N, min. (% TST)	167.5 (47.3)	112 (34.2)
Stage 3N, min. (% TST)	36 (10.2)	24 (7.3)
Stage REM, min. (% TST)	66 (18.6)	106.3 (32.5)
Sleep latency, min.	4.5	11
Latency stage 2, min.	7	14.5
Latency stage REM, min.	5	19.5
Sleep efficiency, %	73.5	67.4
AHI	3.1	2.6
WASO	123.5	147

**Min.**, minutes; **TST**, total sleep time; **REM**, rapid eye movement; **AHI**, Apnea-Hypopnea Index; **WASO**, wake after sleep onset. Sleep efficiency is the ratio of sleep time to time spent in bed, expressed in %.

## Data Availability

Due to restrictions, the data supporting this publication are available upon reasonable request from the corresponding author.
